# Different roles for ipsilateral positive feedback and commissural inhibitory networks in oculomotor velocity to position neural integration

**DOI:** 10.1186/1471-2202-16-S1-P100

**Published:** 2015-12-18

**Authors:** Keiichiro Inagaki, Yutaka Hirata

**Affiliations:** 1 Department Robotic Science and Technology, Chubu University, Kasugai, Aichi, 487-8501, Japan

## 

Saccadic eye movements are made about twice a second to shift our gaze either consciously or unconsciously. Brainstem burst neurons trigger each saccade with a bursting activity closely related to saccadic velocity that must be transformed into persistent firing activity to maintain post-saccadic eye position. A conceptual neural mechanism achieving this velocity to position integration is the oculomotor neural integrator (NI). Neurons in the nucleus of prepositus hypoglossi (NPH) in mammals and equivalently Area I (AI) in goldfish have been demonstrated to exhibit persistent activity relating to eye position. Ipsilateral positive feedback found in NPH and AI [1], and commissural inhibition between these bilateral areas [2] are proposed to be the two candidate neuronal networks for the integration. However, how these networks contribute to generate persistent firing is still unknown. Recent behavioral and optogenetic experiments demonstrated that eye position holding properties are individually modifiable for nasal and temporal hemi-fields of the eye, thereby suggesting existence of at least two NIs in each nucleus [3,4]. In the present study, we constructed a NI neuronal network model closely following anatomical and physiological evidence to better understand the roles of the two NI networks.

The model consists of an excitatory burst neuron (EBN), an inhibitory burst neuron (IBN), a tonic neuron (TN), and a bilateral NI network in which 15 integrator neurons (INs) are included unilaterally. Each IN receives input from TN, EBN, and IBN, and also has positive recurrent feedback connections from all ipsilateral INs including commissural inhibitory connections from all contralateral INs. The output of EBN, IBN, and each NI are weighted-summed at a motor neuron whose output is sent to an eye plant model to simulate eye movements. These neuron models are described as conductance-based spiking neurons. Synaptic weights in the model were carefully tuned to reproduce saccades and post-saccadic stable eye positions. Following individual modification of either the synaptic weights of ipsilateral feedback connection or those of commissural inhibition we measured post-saccadic eye position drift.

Simulation results showed that persistent firing activity of INs is maintained principally by the ipsilateral positive feedback. By contrast, commissural inhibition contributed mainly to null eye position which is the asymptote from which the NI becomes leaky. These findings are the first demonstration by a NI neuronal network model assigning specific and different roles to the ipsilateral positive feedback and the commissural inhibition. In future work, we will use this model to evaluate the elective eye holding properties within the two hemi-field NIs.
